# The Effect of Changes in Physical Self-Concept through Participation in Exercise on Changes in Self-Esteem and Mental Well-Being

**DOI:** 10.3390/ijerph18105224

**Published:** 2021-05-14

**Authors:** Inwoo Kim, Jihoon Ahn

**Affiliations:** 1 Department of Sports Culture, Dongguk University, Seoul 04620, Korea; iwkim@dongguk.edu; 2 Department of Physical Education, Seoul National University, Seoul 08826, Korea

**Keywords:** exercise participation, physical self-concept, self-esteem, mental well-being

## Abstract

The aim of the present study was to examine the impact of the changes in physical self-concept induced by exercise participation on the changes in global self-esteem and mental well-being using a structural model analysis. A total of 189 university students in Seoul, Korea, participated in the present study for two waves. The participants responded through a survey measuring physical self-concept, self-esteem, and mental well-being before and after a six-week exercise course. Regression analysis was used to calculate the amount of change in each variable, and the calculated residual scores were used for correlation analysis and structural model analysis. The amounts of changes in the variables are significantly correlated with each other and there was a complementary mediating effect of the changes in self-esteem on the pathway from the changes in physical self-concept to the changes in mental well-being. Physical self-concept changed by exercise participation might directly and positively influence mental well-being, and it can indirectly influence the changes in mental well-being via the improvement of self-esteem.

## 1. Introduction

Physical activity and exercise are known to be beneficial to health through preventing diseases, enhancing physical abilities, diminishing depression, and promoting happiness [1]. Positive psychologists who have focused on psychological and subjective well-being also emphasized that exercise is one of the important factors in a happy life [2], which has led to a growing interest in the relationship between physical activity and happiness.

Even though physical activity is widely known to be an important factor in reducing negative emotions and leading to a happy life, many people still do not exercise. In particular, the lack of exercise among young adults has been remarkable. According to a study, the level of physical activity among university students was lower than that of adolescents [3], and there is also research showing that the sedentary lifestyle of college students limits the amount of physical activity and causes many health problems [4]. This period is the transition from adolescence to adulthood, which is a very important period for the formation of health behaviors that can be maintained throughout life [5]. Therefore, it is important to encourage university students to participate in sports by emphasizing that exercise is a crucial element of a happy life.

According to existing research related to the correlation between well-being and exercise participation, physical activity can positively influence mental health by reducing negative depression and anxiety and enhancing self-esteem and self-concept [6]. Especially physical self-concept as an important element of the global self-concept has been widely researched in the fields of sports and exercise psychology related to exercise and well-being.

Physical self-concept is defined as one’s perception or evaluation of their physical ability and physical appearance, and it is one of the sub-factors of the global self-esteem with social self-concept and emotional self-concept [7]. Fox and Corbin [7] developed the Physical Self-Perception Profile (PSPP), and identified four sub-factors of the PSPP, including sports competence, attractive body, physical strength, and physical condition. Marsh, Richards, Johnson, Roche and Tremayne [8] redefined the PSPP in terms of eleven subdomains by dividing the four factors in the PSPP and adding self-esteem and general physical self-concept. They developed the Physical Self-Description Questionnaire (PSDQ). Thus, physical self-concept is explained by several sub-factors and it explains one’s general self-perception at the same time in three levels of hierarchical structure.

Many studies have reported that the physical self-concept is positively related with happiness and well-being. Fox [9,10] identified that self-perception of body is essential for mental health and well-being, and Morales-Rodríguez and colleagues [11] reported that physical self-concept is one of the psychosocial factors of the psychological well-being in university students. Roh [12] assured that the perception of one’s physical state is correlated with health perception and psychological well-being, and Kim and Oh [13] also reported by a meta-analysis that there was a positive correlation between physical self-perception and happiness. In other words, existing research has shown that there is a general consensus that developing a positive self-concept is helpful for a happy life.

Although the relationship between physical self-concept and happiness is being discussed from a positive psychological perspective, existing research has been conducted with a limited selection of some elements of happiness, such as psychological well-being, or life satisfaction. However, positive psychologists have defined happiness as a multidimensional concept that includes subjective well-being defined as affirmation and satisfaction of life [14] and psychological well-being [15] pursuing self-actualizing happiness. Moreover, Keyes [16] redefined subjective well-being as emotional well-being and suggested an integrated concept of mental health by adding social well-being to the existing psychological well-being. A recent study insists that, to fully understand mental health, it is necessary to consider the multidimensional nature of well-being, and many existing studies that do not take this into account have missed the key facets of well-being [17]. In other words, the concept of mental health, which includes all aspects of emotional, psychological, and social aspects, is widely applied in studies related to happiness, while existing studies on physical self-concept and well-being did not consider this, so they might have missed important factors in mental health.

To examine the relationship between mental health and physical self-concept, it might be necessary to examine one’s self-esteem. According to early research, developing the physical self-concept could induce the enhancement of one’s self-esteem because it is one of the sub-factors of global self-esteem [7,18]. A study from Garn and colleagues [19] reported that students’ physical self-concept could influence their general self-esteem, and Harther [20] has also supported that physical self-esteem is a major factor influencing global self-esteem. Such implications mean that the change of one’s perception of physical worth could induce positive changes of overall self-perception or self-esteem. 

Physical activity or exercise may play a role in developing physical self-worth. Babic, Morgan, Plotnikoff, Lonsdale, White, and Lubans [21] revealed by a meta-analysis that various perceptions about the body were strongly related to physical activity, and that strategic participation in exercise could develop certain factors of the physical self-concept. In addition, a recent Spanish study identified that physical activity can help adolescents develop a positive self-concept and improve psychological well-being through satisfaction with their body and improvement of their physical self-concept [22]. On the contrary, it was also said that the improvement of self-perception about sports competence and physical fitness can act in the direction of promoting participation in exercise. Thus, it can be said that physical activity can be an antecedent variable of the physical self-concept or an outcome variable [23], which might mean that the causal relationship between the two variables remains ambiguous. This suggests that a longitudinal design, not a cross-sectional, is needed to examine the causal relationship between exercise participation and physical self-concept. 

To sum up, physical activity and exercise are known to contribute to the improvement of happiness and quality of life by positively influencing the physical self-concept and self-esteem. However, despite these advantages, many people still do not exercise, especially university students, who are at a crucial time in the formation of lifelong health behaviors, do not participate enough in exercise. Existing studies have a limitation in that they did not apply the multidimensional concept of happiness defined from positive psychology. Additionally, since the causality of participation in exercise and physical self-concept is unclear, a longitudinal study is needed to examine that the physical self-concept changes through exercise and affects self-esteem and happiness. 

The purpose of the present study was to examine the effect of physical self-concept changed by participation in exercise on the changes in self-esteem and mental health in university students. To consider the multidimensional aspect of happiness, we applied the concept of mental health including emotional well-being, psychological well-being, and social well-being suggested by Keyes [16]. Moreover, a longitudinal design was used to examine the changes of other variables due to participation in the exercise. This study extends the knowledges about how the changes in self-awareness through exercise can affect various aspects of mental health, and through this, it provides implications for the importance and direction of participation in exercise for young students’ happy life.

## 2. Methods

### 2.1. Participants

A total of 189 students from a university located in Seoul, Korea, who are enrolled in physical fitness classes participated in this study. The participants were sampled through the convenient sampling method, and only the students who did not exercise at all outside of class participated in the survey. There were three classes taught by three lecturers but the teaching methods and the contents of all classes consisted equally with various kinds of exercises, such as weightlifting, aerobic exercise, core training, and stretching. The students’ majors varied, but none of them majored in physical education or sports science, and they had no experience taking physical fitness classes in the past. The survey was conducted twice before and after the classes, and 161 students participated in both surveys (102 males and 59 females; M age = 21.8, SD age = 2.1). There were no significant differences between classes and gender in the means of each variable.

### 2.2. Study Procedure

Among the students who voluntarily enrolled in the physical fitness classes, the students who did not exercise outside of the class were encouraged to participate in the study. Prior to conducting the survey, permissions were obtained from the instructor and the internal review board of the researchers’ university, and the questionnaire was distributed only to students who voluntarily agreed to participate in the survey. The first survey was conducted before starting the classes, and the second survey was conducted after the physical fitness classes, which were conducted twice a week for 6 weeks and 100 min per session. The questionnaire was composed of questions that measure physical self-concept, self-esteem, and mental health, and it takes about 15 min to complete. 

### 2.3. Measures

Physical self-concept. The Korean version of the Physical Self-Description Questionnaire (K-PSDQ) [24] translated from the original version developed by Marsh, Richards, Johnson, and Roche [8] was used to measure the participants’ physical self-concept. The K-PSDQ consists of 4 questions per factor, a total of 40 questions for 10 factors, but only four factors that can change through physical fitness classes were selected and used in the study, including strength, endurance, flexibility, and sports competence. Examples of the questions for each factor include: “I am stronger than most people my age” for strength, “I can run a long way without stopping” for endurance, “I am quite good at bending, twisting, and turning my body” for flexibility, and “I am good at sports” for sports competence. A confirmatory factor analysis on the four-factor model with 16 items was conducted, and the result revealed that the model fit was acceptable with the fit indices (χ^2^ = 186.546, df = 98, *p* < 0.001, CFI = 0.962, TLI = 0.954, RMSEA = 0.076). Each item was coded on a 6-point Likert scale (1 = absolutely not, 6 = very much so). The internal consistency of the scale was also acceptable, with the Cronbach’s alpha coefficients ranging from 0.881 to 0.946. 

Self-esteem. To measure self-esteem of the participants, the Korean version of the Rosenberg Self-Esteem Scale (K-RSE) was used [25]. The K-RSE consists of 9 items with 2 factors, excluding item 8 from the original scale, which has been verified as inappropriate due to cultural differences in several studies [25]. Each question was rated on a 4-point Likert scale ranging from 1 (not at all) to 4 (very much), and negative items were reverse-coded so that a high score means high self-esteem. As a result of the confirmatory factor analysis, the construct validity of the two-factor model of the K-RSE was verified with the fit indices (χ^2^ = 54.758, df = 26, *p* = 0.001, TLI = 0.952, CFI = 0.965, RMSEA = 0.083), and Cronbach’s alpha coefficients were 0.87 and 0.875. 

Mental health. The Korean version of the Mental Health Continuum-Short Form (K-MHC–SF) was used to measure the participants’ mental health [26]. The K-MHC–SF is a 14-item scale consisting of three factors: emotional well-being (3 items), social well-being (5 items), and psychological well-being (6 items). Examples of the questions for each factor include: “I felt a sense of happiness” (emotional well-being), “I felt that our society was becoming a better place to live” (social well-being), “I felt that my life had a sense of direction or meaning” (psychological well-being), and each question was answered with the frequency experienced over the past month coded on a 6-point Likert scale (1 = not at all, 2 = once or twice, 3 = about once a week, 4 = about two or three times a week, 5 = almost every day, 6 = every day). As a result of conducting a confirmatory factor analysis on 14 items of 3 factors to verify the construct validity of the scale, two items for social well-being with a standardized regression coefficient of less than 0.5 were deleted. The results of the second confirmatory factor analysis for 12 items revealed that the construct validity was acceptable (χ^2^ = 105.036, df = 51, *p* < 0.001, TLI = 0.944, CFI = 0.956, RMSEA = 0.081), and the internal consistency of the scale was also acceptable, with the Cronbach’s alpha coefficients ranging from 0.839 to 0.935.

### 2.4. Analysis

In this study, the AMOS 18.0 and SPSS 22.0 programs were used to analyze the collected data. First, a descriptive statistical analysis was conducted to understand the overall trends and characteristics (mean and standard deviation, etc.) of the participants. Surveys were conducted twice at intervals of six weeks, and to analyze the individual level of changes in the three variables (physical self-concept, self-esteem, and mental health), residual scores were generated and used for the main analysis. Based on the suggestion from Smith and Beaton [27], after inputting the measurement values in the first wave to the independent variable of the regression equation and the measurement values in the second wave to the dependent variable, the standardized residuals were calculated by regression analysis. Pearson’s correlation analysis was performed to examine the correlations between the standardized residuals, which mean the amount of change in each variable. 

Structural equation modeling was used to examine the structural relationship between the amount of change in the variables. Specifically, we tried to test a model in which changes in self-esteem mediate the relationship between changes in physical self-concept and changes in the three well-beings of mental health. To verify the mediating effect, we used the χ^2^ difference test and bootstrapping, which is a method to verify the confidence level of the indirect effect. At this time, the confidence interval was set to 95% and the number of repetitions was set to 2000.

## 3. Results

### 3.1. Descriptive Statistics 

Table 1 shows the results of descriptive statistics for the general tendency and normality of the variables measured in this study. Although some SD values were less than 1.0, the skewness and kurtosis were below the reference value (≤2, ≤7, respectively) confirming the normality of the data [28]. The means of all variables were higher in the second wave than in the first wave, and as a result of the paired sample *t*-test, all the differences were statistically significant.

### 3.2. Correlations between the Amount of Change in the Measured Variables

The correlations between the amount of change in variables were analyzed and represented in Table 2. As mentioned above, the standardized residuals of the measurement variables were generated and used as the amount of change in each variable. Table 2 shows that flexibility had a significant correlation with all variables except negative self-esteem and emotional well-being, and muscle strength was significantly correlated with all variables except negative self-esteem. All other variables were found to have a significant correlation between each other. 

### 3.3. Testing the Adequacy of the Research Model

**Figure 1:**
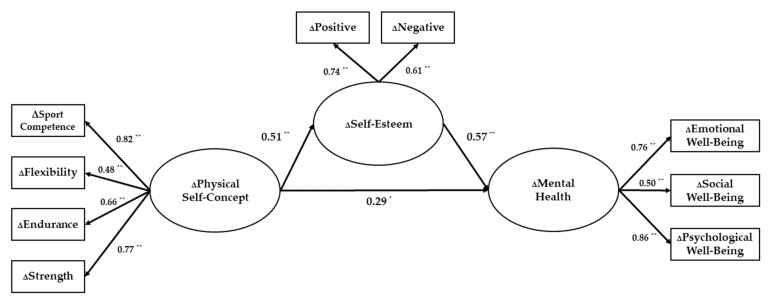
Relationship between change in physical self-concept, change in self-esteem, and change in mental health, * *p* < 0.05, ** *p* < 0.01.

The structural equation model analysis was conducted in which the relationship between changes in physical self-concept and changes in mental health is mediated by the amount of change in self-esteem (Figure 1). The research model showed an acceptable fit to the data with the model fit indices (χ^2^ = 39.805, df = 24, *p* < 0.05, TLI = 0.946, CFI = 0.964, RMSEA = 0.064, SRMR = 0.046). Each path coefficient is shown in Figure 1. 

It was identified that the amount of change in physical self-concept significantly predicted the amount of change in self-esteem (*β* = 0.506, *p* < 0.01), which is the mediating variable, and the amount of change in mental well-being (*β* = 0.287, *p* < 0.05), which is the dependent variable. In addition, the amount of change in self-esteem was found to significantly predict the amount of change in mental well-being (*β* = 0.565, *p* < 0.01).

### 3.4. Mediating Effect of the Amount of Change in Self-Esteem

To verify the mediating effect of the amount of change in self-esteem in the relationship between the amount of change in physical self-concept and the amount of change in mental well-being, the complete mediation model was set as a competition model, and a χ^2^ difference test with the research model was conducted. Table 3 shows the results of comparing the fit of the research model and the fit of the fully mediated model constraining the effect of the change in physical self-concept on the change in mental well-being to “0”. 

As shown in Table 3, there was a statistically significant difference in the value of χ^2^ between the research model and the competition model (Δχ^2^ = 5.353, Δdf = 1, *p* = 0.021). This means that the amount of change in self-esteem partially mediated the relationship between the amount of change in physical self-concept and the amount of change in mental well-being. 

To verify the specific mediating effect, an indirect effect analysis through the bootstrapping method was performed in the 95% confidence interval. As shown in Table 4, the indirect effect (*β* = 0.299, *p* = 0.012) of the change of physical self-concept on the change of mental well-being was verified to be significant. 

## 4. Discussion

Physical self-concept is one of the important factors contributing to the overall self-esteem which is known as an essential element of a happy life. Considering that participation in exercise helps form a physical self-concept, it can be concluded that exercise can lead to happiness. Although several studies that partially support the process of participation in exercise leading to well-being have been implied, there has been no case of empirically examining this process in a single study. In addition, existing studies have shown that the concept of happiness is not an integrated perspective. Therefore, this study attempted to infer the effect of the physical self-concept changed through participation in exercise for a certain period on the changed self-esteem and mental well-being in university students by collecting short-term longitudinal data. In addition, this study attempted to discuss happiness from a more integrated perspective by using the concept of mental well-being, including psychological well-being, emotional well-being, and social well-being.

This study calculated the amount of change in physical self-concept, self-esteem, and mental well-being using data collected by repeated measurements before and after a six-week exercise participation. Based on significant correlations between the amount of change of the variables, a structural equation model analysis was conducted to examine the relationships between variables, and the following implications were derived based on the analysis.

As a result of the analysis, the amount of change in the sub-factors of the physical self-concept was found to have a positive correlation with the amount of change in self-esteem, and the amount of change in the physical self-concept was found to positively predict the amount of change in self-esteem. This is consistent with the results of a study by Garn et al. [19] that showed a positive correlation between physical self-concept and total self-esteem of students participating in a physical education class. In addition, it also supports the study from Ryu and Lee [29] who reported that self-esteem can be predicted through the physical self-concept of the elderly participating in physical activities. According to the hierarchical model of Marsh and colleagues [18], global self-esteem is formed through multidimensional self-concepts including physical self-concept. This study is meaningful in that it confirmed the relationship between the amount of change of each variable through longitudinal data, unlike previous studies using cross-sectional data. Thus, the results of this study imply that the more positively the physical self-concept changes through exercise, the more the self-esteem is improved.

The results from this study also revealed that the amount of change in self-esteem and mental well-being were positively correlated, and the path coefficient leading from the amount of change in self-esteem to the amount of change in mental well-being was also significant. These results indicate that the improved self-esteem through exercise participation positively affects changes in mental well-being, which is in line with some related existing studies [29,30,31]. A study reporting that adult women’s participation in sports contributes to the improvement of self-esteem and subjective happiness [32], and another study [33] which revealed that self-esteem of participants in live sports positively predicts psychological well-being are also consistent with the results of this study. However, these studies did not consider the effect of self-esteem changes through exercise on various aspects of happiness because they selected only one of the factors of psychological well-being [29,31,33] or subjective well-being [32]. The results of this study that the positive changes of self-esteem through exercise participation can enhance complete happiness may be helpful in forming a positive attitude toward participation in exercise among college students.

The amount of change in various sub-factors of the physical self-concept, such as sports competence, appearance, endurance, and muscle strength were found to be positively correlated with the change in mental well-being, and the change in physical self-concept positively predicted the change in mental well-being. This result is partially consistent with the results of theoretical studies [10,19], and empirical studies [12,34] identified that the physical self-concept is one of the positive factors predicting life satisfaction. The notable implications of this study are that the psychological, subjective, and social well-being level can be improved as the positive perception of one’s body increases. 

As a result of χ^2^ difference verification and indirect effect analysis, the amount of change in self-esteem was found to complementarily mediate the relationship between the amount of change in physical self-concept and the amount of change in mental well-being. It can be said that this is the result of empirically verifying the hierarchical relationship between physical self-concept and self-esteem shown in Fox and Corbin’s physical self-perception profile [7], Marsh and colleagues’ [18] physical self-concept model, and a related study that self-esteem is one of strong predictors for hedonic and eudemonic well-being [35]. That is, based on the existing hierarchical model, this study empirically found that step-by-step changes in physical self-concept through participation in exercise can lead to changes in mental well-being through changes in self-esteem. In addition, the results of this study, which imply that changes in physical self-concept can have a direct effect on changes in mental well-being, support the conclusions presented in a recent meta-analysis by Kim and Oh [13]. Moreover, it can be said that it complements the limitations of existing studies which were mainly cross-sectional studies.

## 5. Conclusions

This study was conducted to verify the effects of positive changes in physical self-concept through exercise participation on changes in self-esteem and mental health in university students through a short-term longitudinal data analysis and to present the effect and direction of exercise for the pursuit of happiness. Participation in exercise for six weeks led to positive changes in the level of physical self-concept, self-esteem, and mental well-being of college students, and it was verified that there was a complementary mediating effect through the structural relationship analysis between the amount of change of each variable. These results are meaningful in that the causality of the hierarchical relationship leading to participation in exercise, physical self-concept, self-esteem, and mental well-being was verified. Thus, the present study suggests the direction that it is effective to aim for a change in physical self-concept to pursue a happy life through exercise participation for young adults who are in an important period for the formation of healthy behaviors that can be maintained throughout life.

The limitations of this study and suggestions for further research are as follows. First, the participants of this study consist only of college students enrolled in a university physical fitness class. To generalize the research results, it is necessary to recruit participants of a wider range of ages and conduct repeated research. In addition, in this study, since the liberal arts lecture called physical fitness was used as an exercise method, it was not possible to suggest an effective method for improving the physical self-concept in terms of exercise methods, intensity, and frequency. If follow-up studies using various exercise methods are conducted, a more specific direction can be presented to promote well-being through improvement of the physical self-concept.

## Figures and Tables

**Table 1:** Descriptive statistics and paired sample *t*-test.

	Wave 1				Wave 2				*t*
	M	SD	Skewness	Kurtosis	M	SD	Skewness	Kurtosis	
sport competence	3.01	1.13	0.09	−0.72	3.44	1.15	−0.18	−0.52	−5.72 **
flexibility	3.04	1.13	0.12	−0.62	3.36	1.19	0.25	−0.60	−4.12 **
endurance	2.76	1.24	0.33	−0.74	3.17	1.22	0.12	−0.62	−5.96 **
strength	2.78	0.91	−0.02	−0.54	3.25	0.97	−0.10	−0.30	−7.00 **
positive self-esteem	3.14	0.50	−0.01	−0.04	3.24	0.49	−0.25	0.06	−2.84 **
negative self-esteem	3.34	0.62	−0.85	0.21	3.44	0.60	−1.04	1.00	−2.37 *
emotional well-being	4.20	0.87	−0.39	0.59	4.54	0.90	−0.50	1.00	−4.95 **
social well-being	3.38	1.01	0.18	0.05	3.71	1.06	0.03	−0.26	−4.30 **
psychological well-being	4.02	0.89	−0.21	−0.01	4.33	0.93	−0.33	−0.03	−4.73 **

* *p* < 0.05, ** *p* < 0.01.

**Table 2:** Correlation matrix.

	ΔPhysical Self-Concept				ΔSelf-Esteem		ΔMental Health		
	1.	2.	3.	4.	5.	6.	7.	8.	9.
1. sport competence	1								
2. flexibility	0.32 **	1							
3. endurance	0.58 **	0.34 **	1						
4. strength	0.63 **	0.47 **	0.45 **	1					
5. positive self-esteem	0.32 **	0.25 **	0.32 **	0.33 **	1				
6. negative self-esteem	0.23 *	0.04	0.19 *	0.11	0.45 **	1			
7. emotional well-being	0.38 **	0.11	0.28 **	0.26 **	0.39 **	0.36 **	1		
8. social well-being	0.23 **	0.18 **	0.27 **	0.26 **	0.21 **	0.24 **	0.39 **	1	
9. psychological well-being	0.40 **	0.26 **	0.28 **	0.38 **	0.45 **	0.40 **	0.66 **	0.42 **	1

* *p* < 0.05, ** *p* < 0.01.

**Table 3:** Comparison of fit indices between the research model and the competition model.

	χ^2^	df	*p*	TLI	CFI	RMSEA	SRMR
Research model	39.805	24	<0.05	0.946	0.964	0.064	0.046
Competition model	45.159	25	<0.05	0.934	0.954	0.071	0.054

**Table 4:** Direct, indirect, and total effect.

Path	Direct Effect	Indirect Effect	Total Effect
ΔPhysical self-concept	0.506 *	-	0.506 *
→ ΔSelf-esteem			
ΔSelf-esteem	0.565 **	-	0.565 **
→ Δ Mental well-being			
ΔPhysical self-concept	0.287 *	0.286 *	0.573 **
→ Δ Mental well-being			

* *p* < 0.05, ** *p* < 0.01

## Data Availability

The data presented in this study are available on request from the corresponding author.
